# Association of urinary phthalate metabolite concentrations with body mass index and waist circumference: a cross-sectional study of NHANES data, 1999–2002

**DOI:** 10.1186/1476-069X-7-27

**Published:** 2008-06-03

**Authors:** Elizabeth E Hatch, Jessica W Nelson, M Mustafa Qureshi, Janice Weinberg, Lynn L Moore, Martha Singer, Thomas F Webster

**Affiliations:** 1 Department of Epidemiology, Boston University School of Public Health, 715 Albany St., Boston, MA 02118, USA; 2 Department of Environmental Health, Boston University School of Public Heal, 715 Albany St., Boston, MA 02118, USA; 3 Section of Preventive Medicine & Epidemiology, Boston University School of Medicine, Harrison Court (Room B04), Boston, MA 02118, USA; 4 Department of Biostatistics, Boston University School of Public Health, 715 Albany Street, Crosstown Center-3rd Floor, Boston, MA 02118, USA

## Abstract

**Background:**

Although diet and activity are key factors in the obesity epidemic, laboratory studies suggest that endocrine disrupting chemicals may also affect obesity.

**Methods:**

We analyzed associations between six phthalate metabolites measured in urine and body mass index (BMI) and waist circumference (WC) in National Health and Nutrition Examination Survey (NHANES) participants aged 6–80. We included 4369 participants from NHANES 1999–2002, with data on mono-ethyl (MEP), mono-2-ethylhexyl (MEHP), mono-n-butyl (MBP), and mono-benzyl (MBzP) phthalate; 2286 also had data on mono-2-ethyl-5-hydroxyhexyl (MEHHP) and mono-2-ethyl-5-oxohexyl (MEOHP) phthalate (2001–2002). Using multiple regression, we computed mean BMI and WC within phthalate quartiles in eight age/gender specific models.

**Results:**

The most consistent associations were in males aged 20–59; BMI and WC increased across quartiles of MBzP (adjusted mean BMI = 26.7, 27.2, 28.4, 29.0, p-trend = 0.0002), and positive associations were also found for MEOHP, MEHHP, MEP, and MBP. In females, BMI and WC increased with MEP quartile in adolescent girls (adjusted mean BMI = 22.9, 23.8, 24.1, 24.7, p-trend = 0.03), and a similar but less strong pattern was seen in 20–59 year olds. In contrast, MEHP was inversely related to BMI in adolescent girls (adjusted mean BMI = 25.4, 23.8, 23.4, 22.9, p-trend = 0.02) and females aged 20–59 (adjusted mean BMI = 29.9, 29.9, 27.9, 27.6, p-trend = 0.02). There were no important associations among children, but several inverse associations among 60–80 year olds.

**Conclusion:**

This exploratory, cross-sectional analysis revealed a number of interesting associations with different phthalate metabolites and obesity outcomes, including notable differences by gender and age subgroups. Effects of endocrine disruptors, such as phthalates, may depend upon endogenous hormone levels, which vary dramatically by age and gender. Individual phthalates also have different biologic and hormonal effects. Although our study has limitations, both of these factors could explain some of the variation in the observed associations. These preliminary data support the need for prospective studies in populations at risk for obesity.

## Background

The prevalence of obesity has increased dramatically over the last several decades in the United States (U.S.) and Europe, and more recently in developing countries [1]. Although changes in dietary patterns and physical activity are undoubtedly key causal factors [2], there is growing interest in the possibility that endocrine disrupting chemicals, such as phthalates, may affect obesity-related pathways by altering hormone action or gene expression [3,4]. Exposure to endocrine disrupting chemicals can interfere with synthesis, release, transport, metabolism, binding, action, or elimination of natural hormones. Proposed biologic mechanisms that could underlie an association between endocrine disruptors and obesity include alterations in thyroid and steroid hormone function [5,6], and activation of peroxisome proliferator-activated receptors (PPARs) [7] which play a major role in adipocyte differentiation and energy storage [8].

Phthalates are ubiquitous industrial chemicals used as plasticizers, solvents, lubricants, and stabilizers in the manufacture of consumer products such as children's toys, medical equipment and medications, cosmetics, and food packaging [9]. The general population is exposed to phthalates by ingestion of food and water, dermal exposure, and inhalation of polluted air [10]. Once taken into the body, phthalates are quickly metabolized and excreted in urine and feces. Lower molecular weight phthalates, such as diethyl phthalate (DEP), are metabolized primarily to their hydrolytic monoesters during phase I biotransformation, whereas higher molecular weight phthalates, including di-2-ethylhexyl phthalate (DEHP), are further metabolized to more hydrophilic oxidative metabolites. Monoesters and oxidative metabolites can be excreted unchanged or can undergo glucuronidation in a phase II biotransformation to a more water soluble compound [9,10].

Animal studies have found carcinogenic effects, testicular and ovarian toxicity, and hormonal, hepatic, and renal effects following phthalate exposure [9,11-14]. While the results of human studies are not entirely consistent [9], several have suggested anti-androgenic effects [15-17] and one reported thyroid function changes [18], both of which may play a role in obesity and fat distribution. The only study to directly evaluate phthalates and obesity-related outcomes in humans found a positive correlation between several phthalate metabolites and waist circumference and a measure of insulin resistance among adult males[19].

The U.S. Centers for Disease Control and Prevention (CDC) has conducted biomonitoring on random samples of approximately one-third of the total National Health and Nutrition Examination Survey (NHANES) population since 1999. Over 100 environmental chemicals, including phthalate metabolites, have been measured in blood and/or urine, and are publicly available for the years 1999–2002 [10]. In this analysis, we examined the relationship between six urinary phthalate metabolites and body mass index (BMI) and waist circumference (WC) in male and female subjects, aged 6–80 years, who were participants in NHANES during 1999–2002. Because both body size and hormonal processes vary substantially with age and gender, we conducted stratified analyses within eight age/gender subgroups (6–11, 12–19, 20–59, and 60–80 years).

## Methods

NHANES is a nationally representative random sample of the non-institutionalized U.S. civilian population selected using a complex multistage probability sampling design. Since 1999, NHANES has enrolled approximately 5000 subjects each year. In-home interviews and physical examinations in a mobile exam unit are used to assess behavioral and physical risk factors and characteristics. NHANES obtained written informed consent from all participants, and all data are available on the NHANES website in a de-identified form. Details regarding questionnaires, physical examination components, and testing procedures are available elsewhere [20].

A total of 5149 participants, aged 6 and older, had phthalate measurements and data on BMI and WC. We excluded participants who reported dialysis (n = 4), chemotherapy (n = 8), insulin treatment (n = 64), pregnancy (n = 204), or breastfeeding (n = 39) because of associations with BMI or central adiposity. We also excluded 148 subjects over 80 years old as weight loss commonly occurs in the elderly. Finally, we excluded 313 subjects who had missing values on one or more covariates.

### Measurement of phthalate metabolites

Details of laboratory methods for measuring phthalate metabolites are reported elsewhere [21]. Briefly, spot urine samples were frozen at -20°C and shipped to CDC's National Center for Environmental Health for analysis. Metabolites were measured using isotope dilution high-performance liquid chromatography and mass spectrometry. NHANES measured seven phthalate mono-ester metabolites in urine samples during 1999–2002: mono-ethyl phthalate (MEP), mono-(2-ethyl)-hexyl phthalate (MEHP), mono-benzyl phthalate (MBzP), mono-cyclohexyl phthalate (MCP), mono-isononyl phthalate (MNP), mono-n-octyl phthalate (MOP), and mono-butyl phthalate (MBP), which represents the sum of two isomers, mono-n-butyl and mono-isobutyl phthalate. We analyzed four metabolites detectable in at least 80% of the study population: MEP, MEHP, MBP, and MBzP. Several additional metabolites were measured during 2001–2002. We included mono-(2-ethyl-5-hydroxyhexyl) phthalate (MEHHP) and mono-(2-ethyl-5-oxohexyl) phthalate (MEOHP), two oxidative metabolites of MEHP that are detected at higher levels than MEHP [22,23]. Metabolites less than the limit of detection (LOD) were replaced with the LOD divided by the square root of two [10]. A total of 4369 observations were available for the analyses of MEP, MEHP, MBP, and MBzP, and 2286 subjects for the analyses of MEHHP and MEOHP.

### Anthropometric variables

NHANES measured weight, height, and WC during the physical examination according to a standard protocol [24]. We used BMI as a measure of overall obesity and WC as an indicator of central adiposity, which may be more strongly related to health than BMI [25].

### Diet, exercise, and other potential confounding variables

#### Dietary variables

NHANES assessed diet with a 24-hour recall using a computer-assisted dietary interview. We examined the following dietary variables as potential confounders of the relation between phthalates and obesity/central obesity: (1) energy intake; (2) energy-adjusted macronutrient intakes (total fat, saturated fat, carbohydrates, and protein); (3) grams of fiber per 1000 kilocalories; (4) daily servings in each of the five major U.S. Department of Agriculture (USDA) Food Pyramid categories [26] (dairy, fruit, grains, vegetables, and meat); and (5) discretionary fat and added sugars as defined by the USDA Food Pyramid.

#### Physical activity

Physical activity was assessed for subjects aged 12 and older with a standard leisure time activity questionnaire. Estimated energy expenditure was derived by applying metabolic equivalent (MET) levels to reported frequencies of activities. We also investigated confounding effects of physical activity using other variables, including a dichotomous variable for leisure time physical activity; average minutes/day of leisure activity; a variable which asked respondents whether their activity was greater, the same, or less than others their age; and an index which combined several leisure and non-leisure time activity variables.

We estimated sedentary behavior by hours spent watching television, playing video games, and using a computer outside of work. Parents provided information on their children's behavior during the past 24-hour time period; adolescents (aged 12–19) and adults reported their usual daily habits over the past month.

#### Demographic variables

NHANES collected data on race/ethnicity, education, and family income during the home interview. Education level was categorized as less than high school, high school diploma, and more than high school. We used three alternative approaches to examine potential confounding effects of socio-economic status (SES): self-reported total family income; the poverty-income ratio (PIR, a ratio of U.S. Census-defined poverty level to family income); and a dichotomous indicator which combined several socioeconomic variables. The indicator variable first used income, if available, to categorize participants as lower SES if their income was less than or equal to $20,000. If data on income were missing, education was used to classify participants (aged 19 and older) as low SES if they had less than high school education. If both income and education were missing, evidence of food insecurity among parents or children (e.g., use of food stamps, insufficient food for parents or children) was used to classify subjects as low SES.

#### Smoking and alcohol consumption

Information on smoking and alcohol consumption was available for adults aged 20 and older. Based on information reported in the home interview, we classified subjects as current, former, or never smokers. We categorized alcohol consumption habits (during the prior month) as none, < 1 drink per week, 1–<4 drinks per week, 4–<8 drinks per week, or 8 or more drinks per week.

#### Menopausal status and parity

We used questions on menstrual history to categorize women aged 20 and older as pre- or postmenopausal. Women who were still having regular menstrual cycles (not due to hormone replacement therapy) were considered premenopausal. We classified women as postmenopausal if they had not had a menstrual period for ≥ 12 months or had had a hysterectomy. Women whose status was unclear were assigned missing values. Parity was categorized into nulliparous, one live birth, or more than one live birth.

### Statistical analysis

We carried out gender specific analyses within four age strata: 6–11, 12–19, 20–59, and 60–80 years. We used a locally weighted regression smoother (LOESS) [27] to examine the shape of the relationship between each phthalate as a continuous variable and BMI or WC. As these analyses did not identify natural cut-points, we used age/gender specific quartiles of each metabolite as the exposure variables in multivariate models. We also created quartiles based on the overall distribution of each phthalate in the entire population aged 6–80.

We used multiple linear regression models to examine the relationship between phthalate quartiles and BMI and WC controlling for potential confounding variables. We included age, creatinine, race/ethnicity, height, and SES as core covariates in all models. Age was modeled continuously in children aged 6–11 and adolescents aged 12–19, and categorically (by 10 year age group) in adults. As recommended by Barr et al, we included creatinine as an independent variable in the model, instead of using creatinine-adjusted phthalate values [28]. We included race/ethnicity and SES because of relationships with both phthalates [10,29] and BMI and WC [30,31]. As there were no important differences between the three SES variables described above, we selected the dichotomous variable for the final models because it had the fewest missing values. In general, there appeared to be very little confounding by the remaining variables that were evaluated. In order to simplify the presentation and interpretation of the data, other potential confounders were retained in all of the models for which they were available if their inclusion led to meaningful changes in the exposure effect estimates (>10%) or if they were significant predictors in any of the age and gender subgroup models. Variables included in the final models were percent of calories from total fat (<30, 30-<38, 38+), servings of dairy (tertiles), servings of fruits and vegetables (tertiles), METS/month, TV/video/computer use, smoking (aged 20+), menopausal status, and parity (females aged 20+). We also ran models stratifying by menopausal status in women aged 20–80. Neither alcohol consumption nor education had meaningful effects on the phthalate estimates. Tests for trend were performed by treating phthalate category as a linear predictor in the models.

In evaluating the relationship between MEHP and the outcomes, we ran models that included MEHP's oxidative metabolites separately as covariates in addition to the standard model described above. Hauser [32] and Meeker [18] used this method to address potential individual differences in metabolism of DEHP. We also created a variable for the percent of DEHP that was excreted as MEHP, as described by Hauser (%MEHP) [32]. This involved converting the urinary levels of MEHP, MEHHP, and MEOHP to nanomoles per milliliter, dividing MEHP by the sum of MEHP, MEHHP, and MEOHP, and multiplying by 100.

SAS version 9.1 was used for all analyses. We used Proc Surveyreg to calculate adjusted mean differences in BMI and WC accounting for stratification by geography and the proportion of minority populations and clustering within primary sampling units. We adjusted for the covariates representing the over-sampled subgroups rather than using sampling weights; this adjustment is regarded as a good compromise between efficiency and bias [33,34].

## Results

Tables 1 and 2 present summary statistics for measures of obesity, exposure, and covariates by age, for females and males, respectively. Phthalates were detected in most participants; MEP and MBP were detected in greater than 99%, MBzP and MEHHP in 98%, MEOHP in 97%, and MEHP in 81% of the study sample. MEP was detected at the highest levels in all subgroups. Females had higher levels of all metabolites than males. Except for MEP, levels of phthalates were highest in 6–11 year olds. Females had higher mean BMI in all age groups except children, whereas males had higher mean WC measurements. The high proportion of Hispanic and non-Hispanic black adolescents is attributable to over-sampling in that age group.

**Table 1:** Distribution of selected characteristics among females by age, NHANES 1999–2002, phthalate subsample

	**Females**			
**Characteristics**	**6–11 yrs **(n = 327)	**12–19 yrs **(n = 682)	**20–59 yrs **(n = 761)	**60–80 yrs **(n = 348)

Phthalate metabolites, ^1 ^(% above LOD^2^)	*Geometric mean (SD), μg/gm creatinine*			
MEP (99)	137.7 (2.8)	207.3 (3.2)	225.6 (3.3)	218.6 (3.5)
MBP (99)	48.0 (2.3)	30.8 (2.3)	24.6 (2.3)	25.7 (2.4)
MBzP (98)	34.4 (2.8)	17.1 (2.7)	12.8 (2.7)	11.7 (2.7)
MEHP (81)	5.4 (2.8)	3.8 (2.9)	4.0 (2.9)	3.3 (2.9)
MEOHP (97)	27.5 (2.4)	15.0 (2.4)	12.5 (2.7)	12.4 (2.6)
MEHHP (98)	39.6 (2.5)	21.1 (2.6)	18.3 (2.8)	18.4 (2.7)
	*Arithmetic mean (SD)*			
Age (yrs)	8.6 (1.7)	15.2 (2.3)	39.5 (11.0)	68.4 (5.94)
BMI (kg/m^2^)	18.5 (3.9)	24.0 (5.7)	28.9 (7.3)	29.2 (6.2)
Waist circumference (cm)	64.2 (11.1)	80.2 (13.3)	92.8 (15.3)	97.5 (14.1)
Height (cm)	135.3 (13.8)	160.9 (7.2)	161.8 (6.8)	158.6 (6.8)
Creatinine (mg/dL)	109.1 (55.5)	162.0 (87.0)	124.9 (79.5)	92.5 (60.4)
Physical activity (met equiv hrs/month)	N/A	165.4 (331.5)	57.1 (118.1)	40.2 (92.7)
Dietary intake				
% kilocalories from total fat	33.1 (7.5)	31.9 (8.8)	33.5 (10.0)	32.1 (9.5)
Fruit & veg (servings/day)	3.5 (2.5)	4.0 (3.3)	4.1 (2.9)	4.78 (3.2)
Dairy (servings/day)	1.8 (1.3)	1.5 (1.4)	1.3 (1.4)	1.2 (1.4)
	*Column percent*			
Socioeconomic status (% low)	33.9	35.5	26.7	44.5
Race/ethnicity				
African American	33.3	30.9	19.8	18.7
Hispanic	33.9	39.6	30.0	27.0
Whites and others	32.7	29.5	50.2	54.3
Television viewing (% >2.5 hrs/day)	48.9	51.6	36.4	52.9
Smoking (% current)	N/A	N/A	23.3	12.9

^1 ^μg/g creatinine

^2 ^level of detection

**Table 2:** Distribution of selected characteristics among males by age, NHANES 1999–2002, phthalate subsample

	**Males**			
**Characteristics**	**6–11 yrs **(n = 329)	**12–19 yrs **(n = 662)	**20–59 yrs **(n = 895)	**60–80 yrs **(n = 365)

Phthalate metabolites^1 ^(% above LOD^2^)	*Geometric mean (SD), μg/gm creatinine*			
MEP (99)	93.9 (2.9)	137.0 (3.5)	182.1 (4.1)	184.8 (5.5)
MBP (99)	38.0 (2.2)	19.3 (2.2)	15.3 (2.1)	17.5 (2.5)
MBzP (98)	34.7 (2.6)	15.7 (2.8)	10.1 (2.5)	9.5 (3.1)
MEHP (81)	5.5 (3.1)	2.7 (3.0)	3.3 (3.2)	2.5 (2.9)
MEOHP (97)	26.6 (2.4)	12.2 (2.8)	10.6 (2.8)	9.2 (2.7)
MEHHP (98)	39.1 (2.4)	18.2 (2.8)	16.6 (3.0)	13.2 (2.9)
	*Arithmetic mean (SD)*			
Age (yrs)	8.6 (1.6)	15.4 (2.30)	38.8 (11.1)	68.7 (6.0)
BMI (kg/m^2^)	18.7 (4.1)	23.6 (5.5)	27.9 (5.6)	28.1 (4.7)
Waist circumference (cm)	65.3 (12.1)	81.8 (15.1)	97.6 (15.3)	103.6 (12.1)
Height (cm)	135.6 (11.5)	169.3 (11.1)	175.5 (7.9)	172.2 (7.3)
Creatinine (mg/dL)	110.2 (57.2)	176.0 (93.7)	166.8 (91.1)	132.7 (77.0)
Physical activity (met equiv hrs/month)	N/A	217.8 (295.4)	96.4 (196.1)	59.6 (141.3)
Dietary intake				
% kilocalories from total fat	33.2 (7.3)	32.1 (7.8)	31.8 (10.0)	33.1 (9.6)
Fruit & veg (servings/day)	3.8 (2.8)	4.1 (3.9)	5.1 (4.2)	5.0 (3.9)
Dairy (servings/day)	2.1 (1.4)	2.0 (1.8)	1.6 (1.9)	1.4 (1.6)
	*Column percent*			
Socioeconomic status (% low)	35.0	35.5	27.3	35.1
Race/ethnicity				
African American	33.7	29.5	19.7	16.7
Hispanic	35.0	40.0	29.8	26.6
Whites and others	31.3	30.5	50.5	56.7
Television viewing (% >2.5 hrs/day)	53.2	56.2	43.9	48.0
Smoking (% current)	N/A	N/A	31.1	16.4

^1 ^μg/g creatinine

^2 ^level of detection

In general, phthalate metabolites did not appear to be strongly related to diet. There was some evidence of a positive trend between percent of kilocalories from total fat and level of MEHP and MEP. The medians for MEHP increased from 3.3, 3.4, 3.3, 3.6, to 3.7 μg/gm creatinine across quintiles of dietary fat, and for MEP, from 147.5, 153.1, 150.3, 154.3, to 163.8 μg/gm creatinine. There were some suggestive inverse relationships between the level of several metabolites and the percent of kilocalories from carbohydrates. There did not appear to be important associations between phthalates and the other dietary variables.

Phthalate metabolites were moderately or strongly correlated (Table 3). MEP had the weakest correlations (r<0.26–0.38) with other phthalates. MEHP was strongly correlated with the oxidative metabolites of DEHP, MEHHP and MEOHP (r = 0.68), which were highly correlated with each other (r = 0.98). MBP and MBzP were also strongly correlated (r = 0.72). Creatinine was weakly positively correlated with BMI and WC (r = 0.01–0.18); correlations tended to be stronger among females than males except among adolescents (data not shown).

**Table 3:** Spearman correlation coefficients* between phthalate metabolites, NHANES 1999–2002

**MEP**	1	0.38	0.34	0.26	0.32	0.33
**MBP**		1	0.72	0.46	0.62	0.64
**MBzP**			1	0.39	0.58	0.59
**MEHP**				1	0.68	0.68
**MEHHP**					1	0.98
**MEOHP**						1
	**MEP**	**MBP**	**MBzP**	**MEHP**	**MEHHP**	**MEOHP**

* All coefficients are significant at p < 0.001

**Figure 1:**
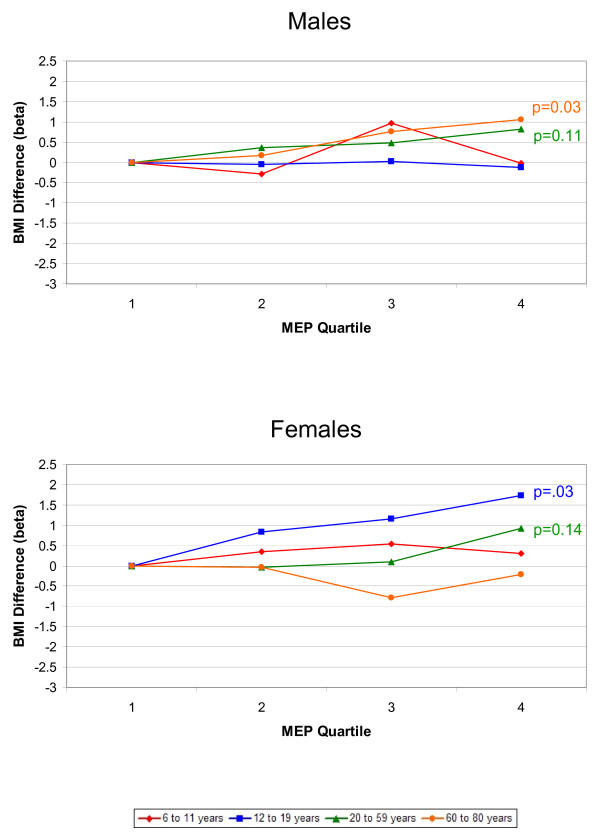
**Difference in Body Mass Index, by Increasing MEP Quartile and Age Group.** Top graph displays results for males, bottom graph for females. All estimates are adjusted for age, race/ethnicity, creatinine, height, SES, dietary factors, TV, Mets/month (age 12+), smoking (age 20+), and reproductive factors (age 20+ females). Results of trend tests are included for those with p-values<0.15.

**Figure 2:**
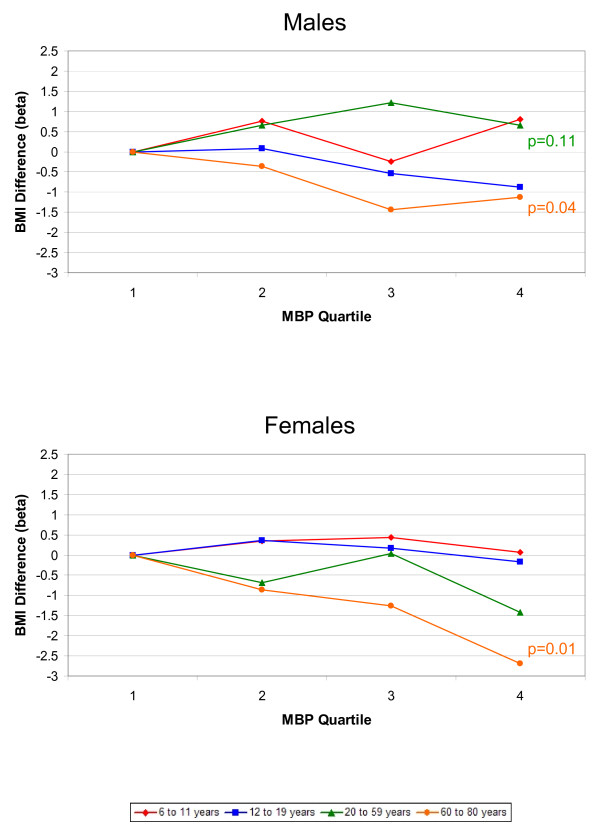
**Difference in Body Mass Index, by Increasing MBP Quartile and Age Group.** Top graph displays results for males, bottom graph for females. All estimates are adjusted for age, race/ethnicity, creatinine, height, SES, dietary factors, TV, Mets/month (age 12+), smoking (age 20+), and reproductive factors (age 20+ females). Results of trend tests are included for those with p-values<0.15.

**Figure 3:**
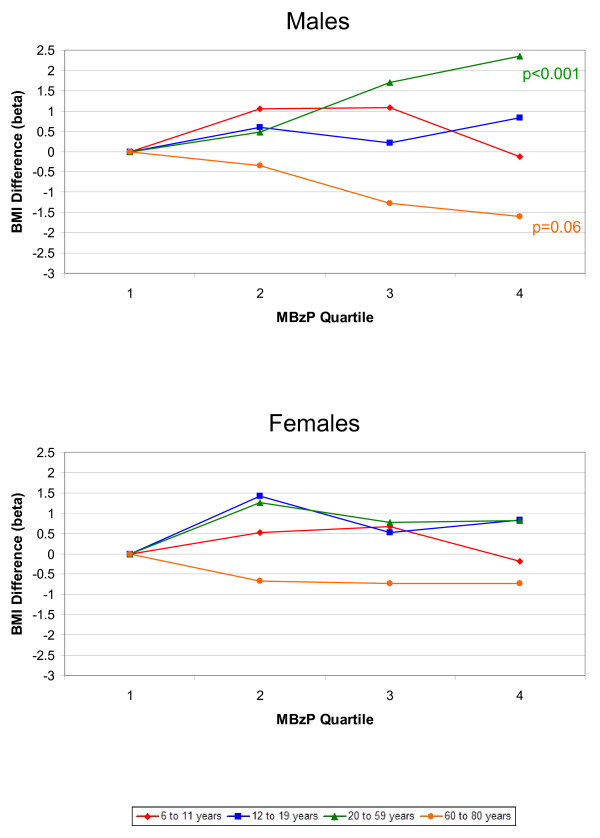
**Difference in Body Mass Index, by Increasing MBzP Quartile and Age Group.** Top graph displays results for males, bottom graph for females. All estimates are adjusted for age, race/ethnicity, creatinine, height, SES, dietary factors, TV, Mets/month (age 12+), smoking (age 20+), and reproductive factors (age 20+ females). Results of trend tests are included for those with p-values<0.15.

**Figure 4:**
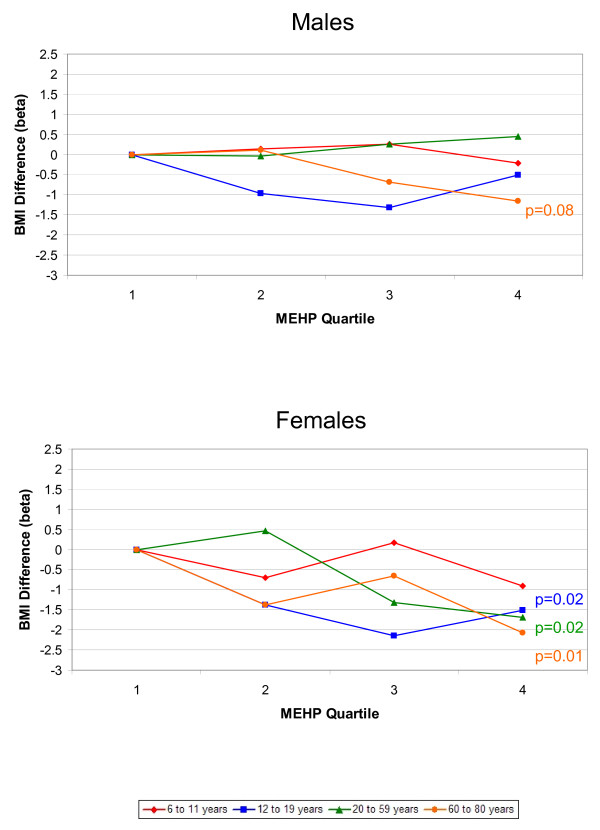
**Difference in Body Mass Index, by Increasing MEHP Quartile and Age Group. **Top graph displays results for males, bottom graph for females. All estimates are adjusted for age, race/ethnicity, creatinine, height, SES, dietary factors, TV, Mets/month (age 12+), smoking (age 20+), and reproductive factors (age 20+ females). Results of trend tests are included for those with p-values<0.15.

**Figure 5:**
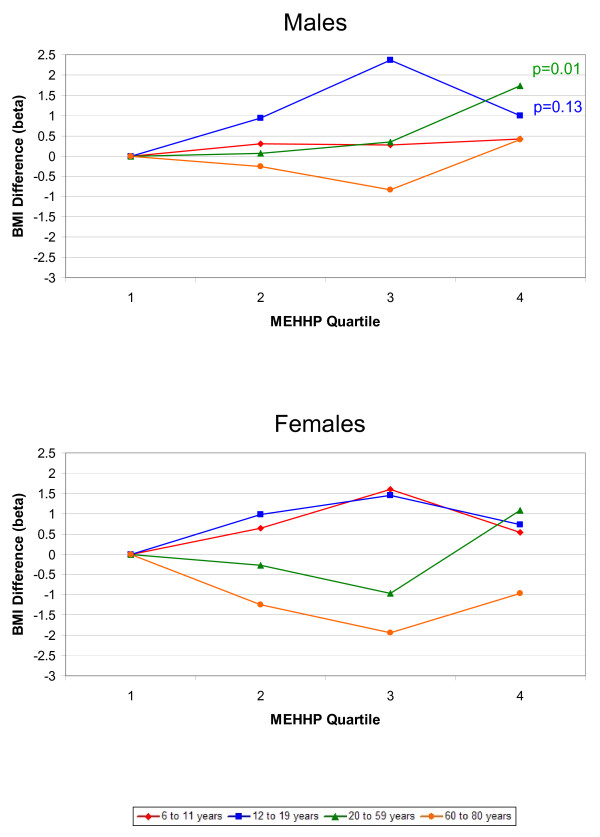
**Difference in Body Mass Index, by Increasing MEHHP Quartile and Age Group.** Top graph displays results for males, bottom graph for females. All estimates are adjusted for age, race/ethnicity, creatinine, height, SES, dietary factors, TV, Mets/month (age 12+), smoking (age 20+), and reproductive factors (age 20+ females). Results of trend tests are included for those with p-values<0.15.

The associations between phthalate metabolites and the outcomes, BMI and WC, were similar; therefore, we only present the results for BMI graphically. Results for both BMI and WC are included in Additional files 1 and 2. Figures 1, 2, 3, 4, 5 present adjusted mean differences in BMI (kg/m^2^) with increasing quartile of phthalate exposure for five metabolites, separately for males and females. Results of trend tests are included for trends with p-values<0.15. The results for MEHHP and MEOHP were virtually identical (MEOHP not shown). We found no major differences in the results when we used cut-points based on quartiles derived from the entire study population; therefore, we present the results for age/gender specific quartiles. We also found no major differences in results when we stratified by menopausal status in adult females (data not shown). Tables 1 and 2 present the ranges for each phthalate quartile, within age and gender subgroups, and the regression coefficients for both BMI (kg/m^2^) and WC (cm) comparing quartiles 2–4 to quartile 1 separately by age, along with 95% confidence limits (CIs) and the p-values for test of trend.

Figure 1 presents results for MEP for males (top) and females (bottom) respectively. A positive relationship between MEP quartile and BMI was apparent for adult males (20–59 and 60–80), and for adolescent and adult (20–59) females. Most of the coefficients for MEP were positive, with the exception of adolescent males (no relationship) and older females (an inverse relationship). For adolescent girls, adjusted mean BMIs were 22.9, 23.8, 24.1, and 24.7 (p-trend = 0.03) with increasing quartile of metabolite concentration, and adjusted mean WCs were 77.4, 79.7, 80.1, and 81.6 (p-trend = 0.02). The effect estimate for WC for the highest quartile of MEP was larger than all other variables in the fully adjusted model; the next largest estimates were -3.1 and -2.9 cm for the top two tertiles of fruit and vegetable consumption, compared to 4.1 and 2.7 cm for the highest two MEP quartiles compared to the lowest. The associations between MEP and BMI and WC in adult women were similar, but less pronounced; the adjusted mean BMIs among women aged 20–59 by MEP quartiles were 28.9, 28.9, 29.0, and 29.8 (p-trend = 0.14). There was some evidence for further effect modification by age with positive effects for 40–59 year old females, but little evidence of an association among 20–39 year olds.

Results for MBP are presented in Figure 2. There were significant inverse trends between MBP level and BMI and WC among 60–80 year olds. There were no clear trends among children or adolescents of either gender. The patterns in adult males and females were different, with suggestive positive trends among males for both BMI and WC, but inverse trends among females.

There was a strong positive relationship between MBzP quartile and BMI and WC among adult males aged 20–59 (Figure 3). The adjusted mean BMIs from quartile 1 to 4 were 26.7, 27.2, 28.4, and 29.0 (p-trend = 0.0002). In contrast, there was a borderline inverse trend among 60–80 year old males. There were no major trends among females (Figure 3), although the estimates were positive among all age groups, except for 60–80 year olds.

MEHP was inversely related to BMI and WC in adolescent and adult females (Figure 4). Mean BMI generally decreased across quartiles among adolescent girls (25.1, 23.7, 23.0, and 23.6 from the lowest to highest quartiles of MEHP, p-trend = 0.02) and among 20–59 year old females (29.8, 30.2, 28.5, and 28.1, p-trend = 0.02). Results were similar among 60–80 year old females. There were no major trends between MEHP and either BMI or WC among males, except for a borderline inverse trend among 60–80 year old males. Results for MEHP were not affected by including either of the oxidative metabolites in the model (data not shown). The percent of DEHP excreted as MEHP ranged from a minimum of 0.3 to a maximum of 98.1%, with medians from 7.8% to 11.8% across the age/gender subgroups. There were no major trends using quartiles of %MEHP as the exposure variable, although most of the coefficients in both males and females were negative, indicating a generally inverse relationship between higher levels of %MEHP and BMI and WC (data not shown).

Figure 5 shows the results for MEHHP, one of the oxidative metabolites of MEHP. There was a positive relationship between MEHHP and BMI among 20–59 year old males, and a non-monotonic increase among 12–19 year old males (Figure 5). For MEHHP, BMI increased from 27.1 to 28.8 between quartiles 1 and 4 (p-trend = 0.10) and WC increased from 95.2 to 99.8 (p-trend = 0.08) among 20–59 year old males. There were no important trends in BMI or WC among females; for 60–80 year olds the estimates were inverse, whereas they were positive for children and adolescents.

## Discussion

This exploratory, cross-sectional analysis of six phthalate metabolites revealed a number of interesting associations with BMI and WC, including several dose-response relationships. Once considered to be a passive organ for energy storage, adipose tissue is now known to be an active endocrine organ that secretes numerous chemical signals and responds to a variety of hormonal signals [35,36] and thus may plausibly be affected by endocrine disrupting chemicals. Animal evidence and limited human data suggest that phthalates have the potential to affect obesity through several different biologic mechanisms that may differ by phthalate (Table 4). These include anti-androgenic effects, inhibition of thyroid hormone action, and activation of PPARs [7,37]. PPARγ in particular has been shown to play a major role in adipocyte differentiation and energy storage [8]; its? activation promotes the differentiation of preadipose cells into adipocytes [37].

**Table 4:** Biological Effects of Phthalates Potentially Related to Obesity

	**PPAR-γ activation**	**Thyroid effect**	**Anti-androgenic effect**
**DEP/MEP**	- [7, 46]	- [47, 48]	+/- [9]
**DBP/MBP**	+/- [7]	+/- [47–51]	++ [9, 16, 52, 53]
**BBzP/MBzP**	+ [7, 37]	++ [47–51]	++ [9, 52, 53]
**DEHP/MEHP**	++ [7, 37, 46, 54, 55]	+/- [18, 47–51]	+ [9, 16, 52, 53]

++ strong, consistent effects

+ moderate, consistent effects

+/- inconsistent effects

- no effect

Our results showed a striking difference in effect between males and females. The strongest positive associations were seen among 20–59 males for MBzP, MEHHP, and MEOHP. In contrast, findings among females varied by metabolite, with some positive associations for MEP, particularly among adolescents, but inverse associations for MEHP and MBzP. Variation in the effect of phthalates by gender is plausible. Several phthalates, as mentioned, are anti-androgens; lower free testosterone levels have been found in males exposed to higher levels of DBP and DEHP [16]. Higher testosterone is associated with smaller WC and a better cardiovascular profile in males, whereas higher androgen levels in females are associated with higher BMI, greater risk for metabolic syndrome, and conditions such as polycystic ovarian disease [38]. Therefore, women with the highest levels of MEHP may have lower levels of androgens or a higher estrogen/androgen ratio, which could explain the inverse relationship between MEHP and BMI. Our results appear somewhat consistent with an anti-androgenic effect of certain phthalates, although additional explanations may be involved, particularly for MEP.

In addition to the gender differences, the associations also differed by age group. We found no associations between phthalates and body size among 6–11 year olds, even though this group had the highest levels of all phthalate metabolites except MEP. In part, this may be due to the smaller sample sizes in this subgroup. BMI and WC are also highly variable in children by age and development status, related in part to timing of the adiposity rebound [39], which could result in less ability to detect effects of external factors. Because metabolite levels are higher in children (except for MEP), the lowest quartile of exposure calculated by age group does not necessarily represent the same low exposure as in other age groups. Even so, results among the 6–11 year olds were substantially the same when exposure quartiles were based on the overall distribution of phthalates in the total study population compared to the results based on the age/gender specific quartiles.

We found mixed associations among adolescents and 20–59 year olds. Different effects by age are plausible because physiology and endogenous hormone levels vary by age in both males and females, potentially modifying the effects of endocrine disruptors. The observed associations among 60–80 year olds were quite different. In general, BMI and WC declined with increasing levels of phthalate metabolites. These results are surprising and deserve further exploration. It is also possible that a physiological change in older people is related to both weight loss and altered phthalate metabolism.

Only one prior study has evaluated the association between phthalates and obesity in humans. Stahlhut [19] also used NHANES data to evaluate the relationship between phthalates and WC, along with a measure of insulin resistance, in adult males. The authors reported similar, mostly positive associations between waist circumference and several phthalates in adult males. Results for children, adolescents, and females were not reported. There were some differences in analytic approach; for example, Stahlhut evaluated all males aged 18 and older, whereas we evaluated 20–59 year olds and 60–80 year olds separately after noticing distinctly different associations in the two groups. We used quartiles of exposure after determining that there were no clear patterns from smoothed regression models, whereas Stahlhut used continuous, log transformed phthalate values, in addition to quintiles of exposure. There were also differences in the confounders that were evaluated in the two studies; in particular, we evaluated a larger number of dietary variables in our models. The qualitative similarity of our results for adult males with those of Stahlhut et al [19] suggest they are reasonably robust to differences in analysis methods. The striking differences between males and females revealed by our analysis significantly add to the earlier work and appears consistent with endocrine disruption by certain phthalates.

Strengths of our study include the broad range of exposures, relatively good statistical power even within subgroups, and detailed evaluation of available potential confounding variables. The ability to evaluate effect modification by age and gender was critical as we saw striking differences in results by these variables. We tested multiple dietary factors, four different measures of physical activity, and three alternative measures of SES. We also evaluated the data using exposure quartiles based on the entire study population in addition to age/gender derived quartiles of exposure, and found that the results were not materially changed.

Our study has several important limitations. Its cross-sectional nature precludes the ability to make any causal inferences about the direction of the association between phthalates and obesity. Although phthalates are rapidly metabolized and do not accumulate in adipose tissue, it is unknown if heavier individuals metabolize phthalates differently than do individuals with less body fat. Elevated exposures to phthalates have been reported due to use of certain medications [40], and it is possible that some of our results may be due to greater use of medications among heavier individuals. It is also possible that our results, particularly for MEP, may be due to greater use of lotions and cosmetics in people with a larger body surface area, resulting in a higher internal and excreted dose. However, due to the multiple environmental sources of DEP, this seems unlikely.

We controlled for numerous confounding variables, including diet and physical activity. However, diet was measured using a 24-hour recall, which has limitations for estimating long-term dietary patterns, and physical activity was measured over the 30 days prior to the interview, which may not reflect long-term patterns. Although NHANES collects data on a wide variety of potential confounders, there may be other unknown or unmeasured confounders. Some of our findings may be due to chance, as we evaluated associations between six phthalate metabolites and two outcomes in eight population sub-groups.

Exposure to mixtures of endocrine disrupting chemicals may have different effects than single exposures [41]. We were unable to examine combined exposures to multiple types of chemicals because NHANES measured chemicals in different population samples. While this dataset does include measurements for multiple phthalates, it is not clear how these exposures should be combined when looking at the relationship between phthalates and body size. Compounds may have different and even potentially opposite effects, and multiple mechanisms may be involved. While work is being done to look at cumulative effects of phthalate exposure on reproductive tract development [42], there currently exist insufficient animal and human data to develop a good biologically-based exposure index for potential effects on obesity.

Single spot-urine measurements of phthalates were used to estimate exposure. Phthalates are rapidly metabolized and excreted, and a single exposure measurement may not reflect long-term exposure. Exposure sources may vary over time based on dietary intake, use of personal care products, medications, and other factors. Several studies have evaluated the consistency of phthalate measurements over time and, in general, low to moderate correlation coefficients were found [43-45], although moderate to high sensitivity to classify individuals into the highest tertile of exposure has been reported [44]. Therefore, our effect estimates are likely affected by non-differential misclassification and comparisons between highest and lowest quartiles may be biased towards the null.

A final limitation of the study is that, due to its cross-sectional nature, we could not evaluate exposures throughout the life course, particularly the sensitive window of prenatal and neonatal development. In utero and early life exposures may be critical; they may permanently alter gene expression patterns that affect metabolic processes [4].

## Conclusion

Our results are exploratory and based on cross-sectional data, but suggest that further research on the potential for phthalates to act as obesogens is warranted. We observed several associations between phthalate metabolites and BMI and WC that were not only statistically significant but of magnitudes of clinical relevance. Prospective studies in human populations are needed to evaluate prenatal and neonatal exposures, and to look at exposure to multiple endocrine disrupting chemicals.

## Abbreviations

Body mass index: BMI; Centers for Disease Control and Prevention: CDC; confidence intervals: (CIs); limit of detection: LOD; locally weighted regression smoother: (LOESS); mono-ethyl phthalate: (MEP); mono-2-ethylhexyl phthalate: (MEHP); mono-n-butyl phthalate: (MBP); mono-benzyl phthalate: (MBzP); mono-2-ethyl-5-hydroxyhexyl phthalate: (MEHHP); mono-2-ethyl-5-oxohexyl phthalate: (MEOHP); National Health and Nutrition Examination Survey: (NHANES); peroxisome proliferator-activated receptors: (PPARs); socioeconomic status: (SES); United States Department of Agriculture: USDA; waist circumference: (WC).

## Competing interests

The authors declare that they have no competing interests.

## Authors' contributions

EEH conceived of, designed, and coordinated the study, oversaw the analysis, and prepared the manuscript. JWN participated in the literature review of phthalate effects, data analysis, and manuscript preparation. MMQ participated in data analysis and manuscript preparation. JW performed statistical analysis and provided statistical expertise. TW and LLM participated in study design, data analysis, and manuscript preparation. MS participated in data set preparation, variable creation, and data analysis. All authors read and approved the final manuscript.

## Supplementary Material

Additional file 1 Quartile ranges for 6 phthalate metabolites, and adjusted* mean difference in BMI (kg/m^2^) and WC (cm) by phthalate quartile, among females by age group, NHANES 1999–2002. Click here for file

Additional file 2 Quartile ranges for 6 phthalate metabolites, and adjusted mean difference in BMI (kg/m^2^) and WC (cm) by phthalate quartile, among males by age group, NHANES 1999–2002. Click here for file
