# Supplementary material for: Association of urinary phthalate metabolite concentrations with body mass index and waist circumference: a cross-sectional study of NHANES data, 1999–2002
Source: Environ Health. 2008 Jun 3;7:27. doi: 10.1186/1476-069X-7-27 (PMC2440739; doi:10.1186/1476-069X-7-27)
Supplement: Additional file 1 — Quartile ranges for 6 phthalate metabolites, and adjusted* mean difference in BMI (kg/m2) and WC (cm) by phthalate quartile, among females by age group, NHANES 1999–2002. [file 1476-069X-7-27-S1.doc]

Quartile ranges for 6 phthalate metabolites, and adjusted* mean difference in BMI (kg/m^2^) and WC (cm) by phthalate quartile, among females by age group, NHANES 1999-2002

| **MEP** | **N** | **Range (μg/L)** | **BMI β (95% CI)** | **p-trend** | **WC β (95% CI)** | **p-trend** |
| --- | --- | --- | --- | --- | --- | --- |
| **Age 6-11** |  |  |  |  |  |  |
| Quartile 4 | 82 | 307.0 - 1836.9 | 0.30 (-1.22, 1.81) | 0.66 | 1.05 (-3.30, 5.40) | 0.61 |
| Quartile 3 | 82 | 129.8 - 305.6 | 0.54 (-0.58, 1.67) |  | 0.99 (-2.05, 4.02) |  |
| Quartile 2 | 82 | 54.8 - 129.6 | 0.35 (-0.86, 1.56) |  | 0.74 (-2.64, 4.12) |  |
| Quartile 1 | 81 | 0.6 - 54.4 | Referent |  | Referent |  |
| **Age 12-19** |  |  |  |  |  |  |
| Quartile 4 | 170 | 694.5 - 39631.7 | 1.74 (-0.02, 3.49) | 0.03 | 4.11 (0.37, 7.86) | 0.02 |
| Quartile 3 | 171 | 265.9 - 694.3 | 1.16 (-0.43, 2.75) |  | 2.7 (-0.83, 6.23) |  |
| Quartile 2 | 171 | 110.8 - 265.7 | 0.84 (-0.59, 2.26) |  | 2.31 (-0.80, 5.42) |  |
| Quartile 1 | 170 | 5.9 - 110.6 | Referent |  | Referent |  |
| **Age 20-59** |  |  |  |  |  |  |
| Quartile 4 | 190 | 512.9 - 21932.2 | 0.92 (-0.51, 2.36) | 0.14 | 2.07 (-0.72, 4.85) | 0.1 |
| Quartile 3 | 191 | 219.5 - 512.0 | 0.10 (-1.02, 1.22) |  | 0.46 (-2.16, 3.07) |  |
| Quartile 2 | 190 | 91.0 - 217.7 | -0.03 (-1.33, 1.26) |  | 0.07 (-2.67, 2.80) |  |
| Quartile 1 | 190 | 1.7 - 90.5 | Referent |  | Referent |  |
| **Age 60-80** |  |  |  |  |  |  |
| Quartile 4 | 86 | 424.0 - 14346.1 | -0.21 (-1.73, 1.32) | 0.64 | -0.22 (-3.49, 3.04) | 0.82 |
| Quartile 3 | 86 | 143.2 - 396.4 | -0.79 (-2.24, 0.66) |  | -1.62 (-4.99, 1.76) |  |
| Quartile 2 | 86 | 56.3 - 142.0 | -0.03 (-1.91, 1.85) |  | -0.62 (-4.29, 3.06) |  |
| Quartile 1 | 86 | 3.4 - 55.6 | Referent |  | Referent |  |
|  |  |  |  |  |  |  |
| **MBP** | **N** | **Range (μg/L)** | **BMI β (95% CI)** | **p-trend** | **WC β (95% CI)** | **p-trend** |
| **Age 6-11** |  |  |  |  |  |  |
| Quartile 4 | 82 | 92.2 - 2595.3 | 0.07 (-1.12, 1.27) | 0.55 | 0.37 (-2.67, 3.40) | 0.84 |
| Quartile 3 | 82 | 51.0 - 92.0 | 0.43 (-0.90, 1.77) |  | 0.69 (-2.74, 4.12) |  |
| Quartile 2 | 82 | 24.7 - 50.8 | 0.35 (-0.75, 1.45) |  | 0.63 (-2.39, 3.64) |  |
| Quartile 1 | 81 | 0.6 - 24.5 | Referent |  | Referent |  |
| **Age 12-19** |  |  |  |  |  |  |
| Quartile 4 | 172 | 84.1 - 1192.9 | -0.17 (-2.24, 1.90) | 0.2 | -0.47 (-4.71, 3.77) | 0.31 |
| Quartile 3 | 169 | 46.5 - 83.7 | 0.17 (-1.60, 1.94) |  | 0.38 (-3.46, 4.23) |  |
| Quartile 2 | 171 | 22.3 - 46.3 | 0.37 (-1.20, 1.93) |  | 1.08 (-2.05, 4.22) |  |
| Quartile 1 | 170 | 0.6 - 22.1 | Referent |  | Referent |  |
| **Age 20-59** |  |  |  |  |  |  |
| Quartile 4 | 191 | 51.7 - 4693.3 | -1.43 (-3.37, 0.52) | 0.29 | -2.60 (-6.15, 0.95) | 0.24 |
| Quartile 3 | 189 | 26.7 - 51.6 | 0.04 (-1.82, 1.90) |  | -0.06 (-3.33, 3.21) |  |
| Quartile 2 | 191 | 11.7 - 26.7 | -0.68 (-1.78, 0.41) |  | -0.61 (-2.87, 1.65) |  |
| Quartile 1 | 190 | 0.6 - 11.6 | Referent |  | Referent |  |
| **Age 60-80** |  |  |  |  |  |  |
| Quartile 4 | 87 | 42.0 - 639.1 | -2.69 (-4.54, -0.84) | 0.01 | -5.67 (-9.31, -2.03) | 0.01 |
| Quartile 3 | 87 | 19.0 - 41.7 | -1.26 (-2.70, 0.18) |  | -3.94 (-7.47, -0.41) |  |
| Quartile 2 | 87 | 9.3 - 18.9 | -0.87 (-2.70, 0.96) |  | -1.85 (-6.19, 2.50) |  |
| Quartile 1 | 87 | 0.6 - 9.3 | Referent |  | Referent |  |
|  |  |  |  |  |  |  |
| **MBzP** | **N** | **Range (μg/L)** | **BMI β (95% CI)** | **p-trend** | **WC β (95% CI)** | **p-trend** |
| **Age 6-11** |  |  |  |  |  |  |
| Quartile 4 | 81 | 68.8 - 1685.0 | -0.18 (-1.43, 1.08) | 0.80 | -0.50 (-3.66, 2.66) | 0.65 |
| Quartile 3 | 83 | 35.7 - 68.3 | 0.68 (-0.50, 1.85) |  | 1.33 (-1.75, 4.41) |  |
| Quartile 2 | 81 | 14.5 - 34.2 | 0.52 (-0.89, 1.92) |  | 1.69 (-1.63, 5.02) |  |
| Quartile 1 | 82 | 0.6 - 14.4 | Referent |  | Referent |  |
| **Age 12-19** |  |  |  |  |  |  |
| Quartile 4 | 170 | 56.9 - 739.7 | 0.84 (-0.97, 2.65) | 0.59 | 1.46 (-3.06, 5.98) | 0.74 |
| Quartile 3 | 171 | 26.9 - 55.9 | 0.53 (-0.96, 2.01) |  | 0.59 (-2.86, 4.05) |  |
| Quartile 2 | 171 | 11.1 - 26.4 | 1.42 (-0.07, 2.91) |  | 2.48 (-0.68, 5.64) |  |
| Quartile 1 | 170 | 0.2 - 11.0 | Referent |  | Referent |  |
| **Age 20-59** |  |  |  |  |  |  |
| Quartile 4 | 190 | 29.8 - 1009.4 | 0.82 (-1.26, 2.90) | 0.62 | 3.18 (-0.90, 7.26) | 0.29 |
| Quartile 3 | 190 | 14.6 - 29.7 | 0.78 (-1.01, 2.56) |  | 2.08 (-1.62, 5.79) |  |
| Quartile 2 | 192 | 5.8 - 14.5 | 1.26 (-0.11, 2.62) |  | 3.55 (0.51, 6.59) |  |
| Quartile 1 | 189 | 0.2 - 5.6 | Referent |  | Referent |  |
| **Age 60-80** |  |  |  |  |  |  |
| Quartile 4 | 87 | 21.8 - 331.9 | -0.73 (-2.67, 1.22) | 0.49 | -2.41 (-6.65, 1.84) | 0.24 |
| Quartile 3 | 88 | 9.1 - 21.6 | -0.72 (-2.85, 1.40) |  | -2.18 (-6.26, 1.91) |  |
| Quartile 2 | 84 | 3.8 - 9.0 | -0.67 (-2.13, 0.80) |  | -1.33 (-5.24, 2.59) |  |
| Quartile 1 | 89 | 0.2 - 3.7 | Referent |  | Referent |  |
|  |  |  |  |  |  |  |
| **MEHP** | **N** | **Range (μg/L)** | **BMI β (95% CI)** | **p-trend** | **WC β (95% CI)** | **p-trend** |
| **Age 6-11** |  |  |  |  |  |  |
| Quartile 4 | 82 | 11.1 - 314.3 | -0.90 (-2.51, 0.71) | 0.45 | -2.51 (-6.52, 1.49) | 0.33 |
| Quartile 3 | 82 | 5.2 - 10.9 | 0.17 (-1.24, 1.59) |  | -0.37 (-3.78, 3.03) |  |
| Quartile 2 | 82 | 2.5 - 5.1 | -0.71 (-1.96, 0.55) |  | -1.76 (-4.69, 1.17) |  |
| Quartile 1 | 81 | 0.7 - 2.4 | Referent |  | Referent |  |
| **Age 12-19** |  |  |  |  |  |  |
| Quartile 4 | 171 | 11.2 - 549.2 | -1.51 (-2.81, -0.21) | 0.02 | -2.18 (-4.99, 0.63) | 0.1 |
| Quartile 3 | 172 | 5.2 - 11.1 | -2.14 (-3.54, -0.74) |  | -3.67 (-6.90, -0.44) |  |
| Quartile 2 | 165 | 2.3 - 5.1 | -1.38 (-2.59, -0.16) |  | -1.99 (-4.60, 0.64) |  |
| Quartile 1 | 174 | 0.7 - 2.2 | Referent |  | Referent |  |
| **Age 20-59** |  |  |  |  |  |  |
| Quartile 4 | 192 | 8.4 - 392.5 | -1.68 (-3.57, 0.21) | 0.02 | -2.17 (-5.99, 1.65) | 0.08 |
| Quartile 3 | 188 | 4.0 - 8.2 | -1.32 (-2.86, 0.22) |  | -2.10 (-5.34, 1.13) |  |
| Quartile 2 | 186 | 1.5 - 3.9 | 0.46 (-1.21, 2.14) |  | 1.97 (-1.69, 5.64) |  |
| Quartile 1 | 195 | 0.7 - 1.4 | Referent |  | Referent |  |
| **Age 60-80** |  |  |  |  |  |  |
| Quartile 4 | 85 | 4.4 - 145.6 | -2.07 (-3.42, -0.73) | 0.01 | -4.15 (-7.48, -0.81) | 0.05 |
| Quartile 3 | 87 | 2.3 - 4.3 | -0.66 (-2.73, 1.42) |  | -0.73 (-5.52, 4.06) |  |
| Quartile 2 | 62 | 1.0 - 2.2 | -1.38 (-3.56, 0.80) |  | -1.89 (-5.85, 2.06) |  |
| Quartile 1 | 114 | 0.7 - 0.8 | Referent |  | Referent |  |
|  |  |  |  |  |  |  |
| **MEHHP** | **N** | **Range (μg/L)** | **BMI β (95% CI)** | **p-trend** | **WC β (95% CI)** | **p-trend** |
| **Age 6-11** |  |  |  |  |  |  |
| Quartile 4 | 43 | 74.5 - 677.6 | 0.54 (-1.50, 2.57) | 0.40 | 1.83 (-3.48, 7.13) | 0.42 |
| Quartile 3 | 43 | 36.8 - 74.4 | 1.60 (-0.53, 3.72) |  | 3.14 (-2.41, 8.68) |  |
| Quartile 2 | 43 | 17.1 - 36.5 | 0.64 (-1.30, 2.57) |  | 1.63 (-2.96, 6.22) |  |
| Quartile 1 | 43 | 0.7 - 16.4 | Referent |  | Referent |  |
| **Age 12-19** |  |  |  |  |  |  |
| Quartile 4 | 85 | 55.0 - 2118.3 | 0.74 (-1.18, 2.65) | 0.33 | 1.81 (-3.19, 6.83) | 0.3 |
| Quartile 3 | 87 | 28.7 - 54.7 | 1.45 (-0.11, 3.00) |  | 3.84 (0.39, 7.29) |  |
| Quartile 2 | 85 | 14.4 - 28.4 | 0.98 (-1.01, 2.97) |  | 1.75 (-2.79, 6.29) |  |
| Quartile 1 | 85 | 0.7 - 14.1 | Referent |  | Referent |  |
| **Age 20-59** |  |  |  |  |  |  |
| Quartile 4 | 99 | 39.3 - 1521.4 | 1.08 (-0.75, 2.92) | 0.29 | 3.13 (-0.73, 6.99) | 0.09 |
| Quartile 3 | 100 | 17.8 - 39.2 | -0.97 (-3.17, 1.23) |  | -0.55 (-4.21, 3.10) |  |
| Quartile 2 | 99 | 7.1 - 17.6 | -0.28 (-2.14, 1.59) |  | -0.46 (-4.03, 3.10) |  |
| Quartile 1 | 100 | 0.7 - 7.0 | Referent |  | Referent |  |
| **Age 60-80** |  |  |  |  |  |  |
| Quartile 4 | 43 | 26.3 - 2227.5 | -0.96 (-4.04, 2.11) | 0.53 | -2.82 (-8.89, 3.25) | 0.38 |
| Quartile 3 | 41 | 13.6 - 25.6 | -1.93 (-5.11, 1.24) |  | -4.82 (-11.51, 1.86) |  |
| Quartile 2 | 44 | 6.7 - 13.4 | -1.24 (-3.53, 1.04) |  | -2.88 (-7.62, 1.86) |  |
| Quartile 1 | 42 | 0.7 - 6.6 | Referent |  | Referent |  |
|  |  |  |  |  |  |  |
| **MEOHP** | **N** | **Range (μg/L)** | **BMI β (95% CI)** | **p-trend** | **WC β (95% CI)** | **p-trend** |
| **Age 6-11** |  |  |  |  |  |  |
| Quartile 4 | 43 | 50.5 - 687.0 | -0.17 (-2.60, 2.26) | 0.79 | 0.45 (-5.56, 6.46) | 0.97 |
| Quartile 3 | 43 | 26.4 - 49.4 | 0.52 (-1.44, 2.48) |  | 0.72 (-4.99, 6.42) |  |
| Quartile 2 | 43 | 11.6 - 24.7 | 0.61 (-1.30, 2.53) |  | 1.52 (-3.28, 6.32) |  |
| Quartile 1 | 43 | 0.8 - 11.1 | Referent |  | Referent |  |
| **Age 12-19** |  |  |  |  |  |  |
| Quartile 4 | 86 | 39.1 - 1380.1 | 0.89 (-1.40, 3.18) | 0.32 | 1.79 (-4.10, 7.68) | 0.37 |
| Quartile 3 | 85 | 20.4 - 38.6 | 0.99 (-0.79, 2.76) |  | 2.62 (-1.64, 6.89) |  |
| Quartile 2 | 86 | 10.7 - 20.3 | 0.43 (-1.74, 2.61) |  | 0.49 (-4.40, 5.39) |  |
| Quartile 1 | 85 | 0.8 - 10.5 | Referent |  | Referent |  |
| **Age 20-59** |  |  |  |  |  |  |
| Quartile 4 | 99 | 28.0 - 914.2 | 0.38 (-1.90, 2.66) | 0.62 | 1.52 (-2.98, 6.02) | 0.38 |
| Quartile 3 | 101 | 12.5 - 27.7 | -0.77 (-3.08, 1.54) |  | -0.18 (-3.84, 3.48) |  |
| Quartile 2 | 99 | 4.9 - 12.3 | -1.03 (-2.28, 0.23) |  | -1.34 (-4.16, 1.49) |  |
| Quartile 1 | 99 | 0.8 - 4.8 | Referent |  | Referent |  |
| **Age 60-80** |  |  |  |  |  |  |
| Quartile 4 | 42 | 17.9 - 1026.4 | 0.94 (-2.98, 4.85) | 0.8 | 2.46 (-7.41, 12.32) | 0.71 |
| Quartile 3 | 43 | 9.3 - 17.5 | -0.76 (-3.70, 2.18) |  | -1.48 (-7.91, 4.95) |  |
| Quartile 2 | 44 | 3.9 - 8.8 | 0.46 (-2.05, 2.97) |  | 0.43 (-6.21, 7.07) |  |
| Quartile 1 | 41 | 0.8 - 3.8 | Referent |  | Referent |  |

* Adjusted for age, creatinine, height, race/ethnicity, socioeconomic status, % of daily calories from total fat (tertiles), daily servings of dairy (tertiles), daily servings of fruit and vegetables (tertiles), METS/month (continuous) (age 12+), TV/video/computer use (< 1 hour/day, >1 and <2.5 hours/day, > 2.5 hours/day), smoking status (age 20+), and menopausal status and parity (women age 20+).
