# Supplementary material for: Association of urinary phthalate metabolite concentrations with body mass index and waist circumference: a cross-sectional study of NHANES data, 1999–2002
Source: Environ Health. 2008 Jun 3;7:27. doi: 10.1186/1476-069X-7-27 (PMC2440739; doi:10.1186/1476-069X-7-27)
Supplement: Additional file 2 — Quartile ranges for 6 phthalate metabolites, and adjusted mean difference in BMI (kg/m2) and WC (cm) by phthalate quartile, among males by age group, NHANES 1999–2002. [file 1476-069X-7-27-S2.doc]

Quartile ranges for 6 phthalate metabolites, and adjusted* mean difference in BMI (kg/m^2^) and WC (cm) by phthalate quartile, among males by age group, NHANES 1999-2002

| **MEP** | **N** | **Range (μg/L)** | **BMI β (95% CI)** | **p-trend** | **WC β (95% CI)** | **p-trend** |
| --- | --- | --- | --- | --- | --- | --- |
| **Age 6-11** |  |  |  |  |  |  |
| Quartile 4 | 82 | 194.4 - 9043.6 | -0.02 (-1.49, 1.46) | 0.65 | -0.67 (-4.42, 3.09) | 0.99 |
| Quartile 3 | 83 | 75.6 - 191.7 | 0.97 (-0.27, 2.20) |  | 1.42 (-1.73, 4.57) |  |
| Quartile 2 | 82 | 39.3 - 75.5 | -0.29 (-1.50, 0.92) |  | -0.75 (-4.01, 2.51) |  |
| Quartile 1 | 82 | 4.7 - 39.0 | Referent |  | Referent |  |
| **Age 12-19** |  |  |  |  |  |  |
| Quartile 4 | 165 | 525.9 - 12359.0 | -0.13 (-1.63, 1.37) | 0.89 | -1.20 (-5.14, 2.74) | 0.64 |
| Quartile 3 | 166 | 195.6 - 524.9 | 0.02 (-1.13, 1.17) |  | -0.30 (-3.49, 2.89) |  |
| Quartile 2 | 166 | 72.9 - 195.3 | -0.05 (-1.23, 1.13) |  | -1.00 (-3.96, 1.96) |  |
| Quartile 1 | 165 | 0.6 - 72.7 | Referent |  | Referent |  |
| **Age 20-59** |  |  |  |  |  |  |
| Quartile 4 | 224 | 698.4 - 39938 | 0.82 (-0.15, 1.79) | 0.11 | 2.19 (-0.51, 4.90) | 0.11 |
| Quartile 3 | 224 | 247.4 - 697.5 | 0.47 (-0.68, 1.63) |  | 1.25 (-1.75, 4.25) |  |
| Quartile 2 | 224 | 80.7 - 245.2 | 0.36 (-0.94, 1.67) |  | 0.85 (-2.44, 4.15) |  |
| Quartile 1 | 223 | 0.6 - 80.4 | Referent |  | Referent |  |
| **Age 60-80** |  |  |  |  |  |  |
| Quartile 4 | 91 | 799.5 - 16995.2 | 1.05 (-0.10, 2.21) | 0.03 | 1.68 (-1.75, 5.10) | 0.21 |
| Quartile 3 | 92 | 177.7 - 787.5 | 0.76 (-0.54, 2.06) |  | 1.55 (-2.11, 5.21) |  |
| Quartile 2 | 91 | 52.1 - 175.4 | 0.18 (-1.21, 1.56) |  | 0.11 (-3.62, 3.85) |  |
| Quartile 1 | 91 | 0.6 - 51.5 | Referent |  | Referent |  |
|  |  |  |  |  |  |  |
| **MBP** | **N** | **Range (μg/L)** | **BMI β (95% CI)** | **p-trend** | **WC β (95% CI)** | **p-trend** |
| **Age 6-11** |  |  |  |  |  |  |
| Quartile 4 | 82 | 71.4 - 638.5 | 0.80 (-0.42, 2.03) | 0.56 | 1.25 (-1.91, 4.40) | 0.86 |
| Quartile 3 | 83 | 37.9 - 70.7 | -0.24 (-1.91, 1.42) |  | -1.28 (-5.74, 3.18) |  |
| Quartile 2 | 83 | 18.5 - 37.8 | 0.77 (-0.37, 1.90) |  | 1.24 (-1.72, 4.19) |  |
| Quartile 1 | 81 | 1.5 - 18.4 | Referent |  | Referent |  |
| **Age 12-19** |  |  |  |  |  |  |
| Quartile 4 | 165 | 54.6 - 969.9 | -0.87 (-2.54, 0.79) | 0.2 | -1.47 (-5.41, 2.48) | 0.31 |
| Quartile 3 | 166 | 31.2 - 54.4 | -0.53 (-1.77, 0.70) |  | -0.70 (-4.02, 2.62) |  |
| Quartile 2 | 166 | 16.5 - 31.1 | 0.09 (-1.32, 1.49) |  | 0.83 (-2.78, 4.43) |  |
| Quartile 1 | 165 | 0.6 - 16.4 | Referent |  | Referent |  |
| **Age 20-59** |  |  |  |  |  |  |
| Quartile 4 | 223 | 41.6 - 397.1 | 0.65 (-0.39, 1.69) | 0.11 | 2.91 (0.22, 5.60) | 0.01 |
| Quartile 3 | 226 | 23.4 - 41.3 | 1.22 (0.35, 2.09) |  | 3.67 (1.27, 6.07) |  |
| Quartile 2 | 224 | 11.3 - 23.3 | 0.66 (-0.48, 1.79) |  | 1.86 (-1.05, 4.77) |  |
| Quartile 1 | 222 | 0.6 - 11.3 | Referent |  | Referent |  |
| **Age 60-80** |  |  |  |  |  |  |
| Quartile 4 | 91 | 38.4 - 4363.4 | -1.12 (-2.49, 0.24) | 0.04 | -2.60 (-6.05, 0.85) | 0.08 |
| Quartile 3 | 91 | 20.2 - 38.3 | -1.44 (-2.61, -0.28) |  | -2.60 (-5.27, 0.07) |  |
| Quartile 2 | 92 | 10.3 - 19.8 | -0.36 (-1.79, 1.07) |  | -0.65 (-4.09, 2.80) |  |
| Quartile 1 | 91 | 0.6 - 10.0 | Referent |  | Referent |  |
|  |  |  |  |  |  |  |
| **MBzP** | **N** | **Range (μg/L)** | **BMI β (95% CI)** | **p-trend** | **WC β (95% CI)** | **p-trend** |
| **Age 6-11** |  |  |  |  |  |  |
| Quartile 4 | 82 | 77.0 - 721.9 | -0.13 (-1.53, 1.28) | 0.80 | 0.55 (-3.31, 4.40) | 0.85 |
| Quartile 3 | 83 | 35.0 - 75.6 | 1.09 (-0.36, 2.54) |  | 2.42 (-1.43, 6.27) |  |
| Quartile 2 | 82 | 15.3 - 34.9 | 1.05 (-0.60, 2.71) |  | 2.52 (-1.71, 6.74) |  |
| Quartile 1 | 82 | 0.4 - 15.0 | Referent |  | Referent |  |
| **Age 12-19** |  |  |  |  |  |  |
| Quartile 4 | 166 | 49.7 - 1274.3 | 0.84 (-0.47, 2.15) | 0.3 | 3.10 (-0.67, 6.88) | 0.15 |
| Quartile 3 | 164 | 26.0 - 49.5 | 0.21 (-0.85, 1.27) |  | 1.36 (-1.47, 4.19) |  |
| Quartile 2 | 166 | 12.0 - 25.9 | 0.60 (-0.65, 1.86) |  | 2.14 (-0.99, 5.28) |  |
| Quartile 1 | 166 | 0.2 - 11.8 | Referent |  | Referent |  |
| **Age 20-59** |  |  |  |  |  |  |
| Quartile 4 | 223 | 30.9 - 1197.6 | 2.35 (1.04, 3.65) | 0.0002 | 6.63 (3.42, 9.84) | <.0001 |
| Quartile 3 | 223 | 14.8 - 30.8 | 1.70 (0.65, 2.76) |  | 4.87 (2.18, 7.56) |  |
| Quartile 2 | 226 | 7.4 - 14.6 | 0.47 (-0.53, 1.48) |  | 1.27 (-1.34, 3.87) |  |
| Quartile 1 | 223 | 0.2 - 7.3 | Referent |  | Referent |  |
| **Age 60-80** |  |  |  |  |  |  |
| Quartile 4 | 91 | 25.3 - 414.8 | -1.59 (-3.43, 0.24) | 0.06 | -3.18 (-7.64, 1.28) | 0.09 |
| Quartile 3 | 93 | 11.3 - 25.0 | -1.27 (-2.97, 0.42) |  | -1.84 (-5.61, 1.93) |  |
| Quartile 2 | 90 | 4.8 - 11.2 | -0.35 (-1.60, 0.89) |  | -0.11 (-3.65, 3.43) |  |
| Quartile 1 | 91 | 0.2 - 4.7 | Referent |  | Referent |  |
|  |  |  |  |  |  |  |
| **MEHP** | **N** | **Range (μg/L)** | **BMI β (95% CI)** | **p-trend** | **WC β (95% CI)** | **p-trend** |
| **Age 6-11** |  |  |  |  |  |  |
| Quartile 4 | 82 | 11.7 - 196.3 | -0.22 (-1.32, 0.89) | 0.76 | -0.20 (-2.98, 2.57) | 0.96 |
| Quartile 3 | 82 | 5.2 - 11.4 | 0.25 (-0.84, 1.35) |  | 0.39 (-2.76, 3.53) |  |
| Quartile 2 | 84 | 2.2 - 5.1 | 0.14 (-1.06, 1.33) |  | 0.06 (-2.99, 3.11) |  |
| Quartile 1 | 81 | 0.7 - 2.1 | Referent |  | Referent |  |
| **Age 12-19** |  |  |  |  |  |  |
| Quartile 4 | 167 | 8.6 - 273.4 | -0.50 (-1.95, 0.94) | 0.46 | -1.39 (-5.15, 2.37) | 0.44 |
| Quartile 3 | 166 | 3.7 - 8.5 | -1.32 (-2.58, -0.06) |  | -3.43 (-6.86, -0.01) |  |
| Quartile 2 | 165 | 1.8 - 3.6 | -0.97 (-2.24, 0.31) |  | -2.45 (-5.82, 0.91) |  |
| Quartile 1 | 164 | 0.7 - 1.7 | Referent |  | Referent |  |
| **Age 20-59** |  |  |  |  |  |  |
| Quartile 4 | 223 | 10.0 - 615.6 | 0.44 (-0.63, 1.52) | 0.35 | 0.91 (-1.43, 3.24) | 0.44 |
| Quartile 3 | 220 | 4.6 - 9.8 | 0.26 (-1.05, 1.57) |  | 0.02 (-3.39, 3.43) |  |
| Quartile 2 | 231 | 1.9 - 4.5 | -0.04 (-0.92, 0.83) |  | -0.15 (-2.47, 2.17) |  |
| Quartile 1 | 221 | 0.7 - 1.8 | Referent |  | Referent |  |
| **Age 60-80** |  |  |  |  |  |  |
| Quartile 4 | 90 | 5.9 - 204.2 | -1.16 (-2.60, 0.28) | 0.08 | -2.42 (-5.76, 0.93) | 0.16 |
| Quartile 3 | 91 | 2.5 - 5.6 | -0.69 (-2.16, 0.79) |  | -0.57 (-4.27, 3.12) |  |
| Quartile 2 | 78 | 1.0 - 2.4 | 0.11 (-1.39, 1.61) |  | 0.64 (-3.16, 4.45) |  |
| Quartile 1 | 106 | 0.7 - 0.8 | Referent |  | Referent |  |
|  |  |  |  |  |  |  |
| **MEHHP** | **N** | **Range (μg/L)** | **BMI β (95% CI)** | **p-trend** | **WC β (95% CI)** | **p-trend** |
| **Age 6-11** |  |  |  |  |  |  |
| Quartile 4 | 48 | 66.4 - 622.7 | 0.42 (-1.09, 1.92) | 0.57 | 1.27 (-2.43, 4.96) | 0.40 |
| Quartile 3 | 49 | 34.4 - 65.2 | 0.27 (-0.97, 1.52) |  | 1.11 (-2.44, 4.65) |  |
| Quartile 2 | 49 | 19.1 - 34.3 | 0.30 (-1.01, 1.61) |  | 0.45 (-3.37, 4.26) |  |
| Quartile 1 | 48 | 0.7 - 18.7 | Referent |  | Referent |  |
| **Age 12-19** |  |  |  |  |  |  |
| Quartile 4 | 80 | 54.9 - 977.0 | 1.00 (-0.69, 2.69) | 0.13 | 2.15 (-1.77, 6.08) | 0.2 |
| Quartile 3 | 81 | 27.1 - 54.2 | 2.37 (0.62, 4.11) |  | 6.53 (2.23, 10.82) |  |
| Quartile 2 | 80 | 12.9 - 26.9 | 0.94 (-0.57, 2.46) |  | 3.07 (-0.06, 6.19) |  |
| Quartile 1 | 81 | 0.7 - 12.5 | Referent |  | Referent |  |
| **Age 20-59** |  |  |  |  |  |  |
| Quartile 4 | 125 | 48.8 - 3236.1 | 1.74 (-0.28, 3.76) | 0.1 | 4.60 (-0.03, 9.24) | 0.08 |
| Quartile 3 | 126 | 21.6 - 48.7 | 0.35 (-1.17, 1.87) |  | 0.65 (-3.23, 4.52) |  |
| Quartile 2 | 126 | 11.0 - 21.4 | 0.06 (-1.33, 1.46) |  | 0.80 (-2.67, 4.28) |  |
| Quartile 1 | 125 | 0.7 - 10.9 | Referent |  | Referent |  |
| **Age 60-80** |  |  |  |  |  |  |
| Quartile 4 | 46 | 30.8 - 1408.9 | 0.41 (-2.47, 3.28) | 0.85 | 0.68 (-7.42, 8.78) | 0.83 |
| Quartile 3 | 47 | 12.9 - 30.5 | -0.84 (-3.84, 2.16) |  | -0.95 (-8.84, 6.94) |  |
| Quartile 2 | 46 | 7.1 - 12.8 | -0.25 (-2.59, 2.09) |  | -1.10 (-6.97, 4.78) |  |
| Quartile 1 | 47 | 0.7 - 6.3 | Referent |  | Referent |  |
|  |  |  |  |  |  |  |
| **MEOHP** | **N** | **Range (μg/L)** | **BMI β (95% CI)** | **p-trend** | **WC β (95% CI)** | **p-trend** |
| **Age 6-11** |  |  |  |  |  |  |
| Quartile 4 | 48 | 47.2 - 351.6 | 0.14 (-1.21, 1.48) | 0.91 | 0.60 (-2.68, 3.88) | 0.77 |
| Quartile 3 | 50 | 22.9 - 46.5 | -0.09 (-0.99, 0.80) |  | 0.06 (-2.55, 2.66) |  |
| Quartile 2 | 47 | 13.0 - 22.6 | 0.17 (-1.36, 1.70) |  | 0.74 (-3.95, 5.42) |  |
| Quartile 1 | 49 | 0.8 - 12.8 | Referent |  | Referent |  |
| **Age 12-19** |  |  |  |  |  |  |
| Quartile 4 | 81 | 36.1 - 607.1 | 0.27 (-1.40, 1.94) | 0.65 | 0.68 (-2.67, 4.02) | 0.76 |
| Quartile 3 | 79 | 18.5 - 35.8 | 1.74 (-0.08, 3.56) |  | 4.67 (0.11, 9.23) |  |
| Quartile 2 | 82 | 8.8 - 18.3 | 0.92 (-1.14, 2.97) |  | 3.34 (-1.09, 7.78) |  |
| Quartile 1 | 80 | 0.8 - 8.7 | Referent |  | Referent |  |
| **Age 20-59** |  |  |  |  |  |  |
| Quartile 4 | 125 | 31.5 - 926.7 | 2.14 (-0.13, 4.41) | 0.09 | 5.81 (0.69, 10.94) | 0.06 |
| Quartile 3 | 126 | 14.7 - 30.7 | 0.96 (-0.67, 2.60) |  | 1.78 (-1.98, 5.54) |  |
| Quartile 2 | 125 | 7.0 - 14.6 | 0.65 (-0.77, 2.07) |  | 2.18 (-1.15, 5.51) |  |
| Quartile 1 | 126 | 0.8 - 6.9 | Referent |  | Referent |  |
| **Age 60-80** |  |  |  |  |  |  |
| Quartile 4 | 46 | 19.9 - 766.9 | 0.69 (-2.05, 3.44) | 0.75 | 2.31 (-4.97, 9.59) | 0.6 |
| Quartile 3 | 48 | 9.1 - 19.7 | -0.94 (-3.42, 1.55) |  | -1.02 (-7.79, 5.76) |  |
| Quartile 2 | 46 | 4.9 - 9.0 | 0.03 (-1.94, 2.01) |  | 0.22 (-4.93, 5.38) |  |
| Quartile 1 | 46 | 0.8 - 4.8 | Referent |  | Referent |  |

* Adjusted for age, creatinine, height, race/ethnicity, socioeconomic status, % of daily calories from total fat (tertiles), daily servings of dairy (tertiles), daily servings of fruit and vegetables (tertiles), METS/month (continuous), TV/video/computer use (< 1 hour/day, >1 and <2.5 hours/day, > 2.5 hours/day), and smoking status (age 20+)
